# Piecewise Business Bubble System under Classical and Nonsingular Kernel of Mittag–Leffler Law

**DOI:** 10.3390/e25030459

**Published:** 2023-03-06

**Authors:** Chao Zhang, Bo Li

**Affiliations:** 1 School of Management, Hefei University of Technology, Hefei 230002, China; 2 School of Finance, Anhui University of Finance and Economics, Bengbu 233030, China

**Keywords:** piecewise financial bubble model, classical derivative, Atangana–Baleanu operator, existence and uniqueness of solution, simulation of curves

## Abstract

This study aims to investigate the dynamics of three agents in the emerging business bubble model based on the Mittag–Leffler law pertaining to the piecewise classical derivative and non-singular kernel. By generalizing the business bubble dynamics in terms of fractional operators and the piecewise concept, this study presents a new perspective to the field. The entire set of intervals is partitioned into two piecewise intervals to analyse the classical order and conformable order derivatives of an Atangana–Baleanu operator. The subinterval analysis is critical for removing discontinuities in each sub-partition. The existence and uniqueness of the solution based on a piecewise global derivative are tested for the considered model. The approximate root of the system is determined using the piecewise numerically iterative technique of the Newton polynomial. Under the classical order and non-singular law, the approximate root scheme is applied to the piecewise derivative. The curve representation for the piece-wise globalised system is tested by applying the data for the classical and different conformable orders. This establishes the entire density of each compartment and shows a continuous spectrum instead of discrete dynamics. The concept of this study can also be applied to investigate crossover behaviours or abrupt changes in the dynamics of the values of each market.

## 1. Introduction

Our study focuses on the mechanisms of chaos and how they can be leveraged to develop new economic models using the available materials [1]. Specifically, we have discovered that the 3D saddle focusing on equilibrium points can generate spiral strange attractors that can result in unpredictable and unreliable economic behaviour, challenging the ability of an economy to reach a long-term steady equilibrium [2]. In light of these findings, we have identified various equilibria that are related to the starting values of the controlling parameters in the model. These equilibria can result in undesired low-growth equilibrium points, which can hinder economic progress. By understanding the dynamics of chaos and its potential implications for economic policy, we aim to contribute to the development of more robust and resilient economic systems.

In 2008, research was conducted to analyse the economic downturn triggered by the financial bubble in the banking sector. The collapse of the bubble caused the economy to plummet to low levels of equilibrium, resulting in the eventual collapse of the financial sector. The impact on the banking sector was evident in the reduction of household deposits which indirectly affected the lending to manufacturing firms, which resulted in a decline in the final output. The weakened state of the banking sector raised concerns over bank solvency, reduced credit availability, and eroded investor confidence, resulting in a decline in global stock markets.

Macroeconomists are facing a significant challenge in the aftermath of the recent financial crisis. Conventional macroeconomic models have ignored financial frictions and instead assumed the existence of perfect financial markets. Consequently, these models are of limited use in explaining financial crises [3,4,5,6]. Furthermore, these models tend to focus on financial frictions in non-financial firms, while overlooking the critical role of financial intermediation in the recent crisis. To gain a more comprehensive understanding of the crisis and inform policy decisions, macroeconomists must develop new models that account for the intricacies of financial markets. For more details, the readers are requested to review the published studies [7,8,9,10,11].

In this study, we consider a financial bubble model [12,13] that includes the three-dimensional system of differential equations:

(1) $$\begin{array}{cc} \frac{dQ}{dt} & =rQ-Q\left[r_{k}+\left(r_{k}-r\right)\xi Q\right]-\theta \left(1-Q\right),\\ \frac{dB}{dt} & =rB-(r_{k}-r)QB,\\ \frac{dN}{dt} & =\left[r_{k}-\theta +\left(r_{k}-r\right)\xi Q\right]N+\left(r_{k}-r\right)B. \end{array}$$

Let Q denote the shadow price of the net worth of a bank N and B be the bubble component of the stock market value of the bank. Where θ∈(0,1), *r*, and ξ∈(0,1) represent the share of bank dividends, rate of deposits, and degree of financial friction, respectively, and $r_{k}=\alpha {\left(\frac{\theta -r}{r_{k}-r}N\right)}^{\alpha -1}-\vartheta$.

Fractional calculus (FC), an extension of traditional calculus, has recently attracted considerable attention in academia [14,15]. Certain researchers argue that traditional calculus, while widely used as an analytical approach, does not significantly contribute to innovation and can be challenging for use for complex theoretical analyses. In contrast, FC provides a more flexible mathematical framework for solving problems that cannot be easily addressed using integer-order calculus. Introduced in 1967, modern calculus was popularised by Caputo in many fields [16]. However, integer-order dynamics a re limited to integer orders with a clear formulation. In contrast, fractional operators, such as the Caputo derivative, generalise the integer-order operators and are essential for solving many practical problems [17,18,19,20]. Researchers have tested various models using the Caputo framework to develop more accurate and robust fractional operators. Caputo and Fabrizio further modified the Caputo operator by introducing a non-singular kernel, and in 2016, Atangana and Baleanu proposed a new definition based on Caputo’s concept using a non-local and non-singular kernel [21,22]. Since then, researchers have conducted considerable research on these operators, exploring various mathematical properties and applications in various fields [23,24,25,26,27,28,29,30].

In a recent study, Araz and Atangana proposed a novel operator for a piecewise differential and integral [31]. The piecewise derivative is divided into two subintervals, where one subinterval is analysed using one fractional operator, and the other is analysed using a different fractional operator. This approach allows for greater flexibility and accuracy while dealing with complex systems with multiple characteristics. To address the issue of crossover behaviour dynamics, the researchers introduced a novel piecewise derivative method [31]. This new approach has been the subject of considerable discussion in the research community, with researchers exploring its potential applications in various fields. Several applications of fractional operators have already been reported in the literature [32,33,34,35,36,37]. These applications range from modelling complex physical systems, such as electrical circuits, to analysing and controlling biological processes. The versatility and effectiveness of fractional operators have rendered them an increasingly popular tool in the mathematical modelling of complex systems [38,39,40].

Using the new operator mentioned above, we analyse the model described in Equation (1) based on the non-singular Mittag–Leffler law and an integer order approach. This analysis provides valuable insights into the behaviour of the system and reveals its underlying dynamics.

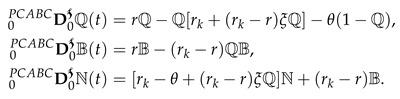
(2)
where PCABC represents the piecewise classical and Atangana–Baleanu derivative with two subintervals in [0,T]. In the simplest form, we can write Equation (2) as

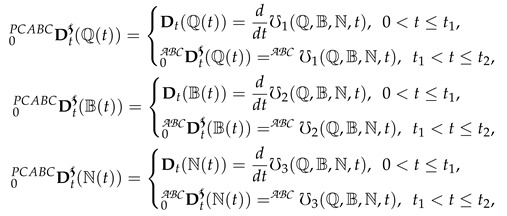
(3)
where 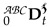 and $D_{t}$ represent the Atangana—Baleanu and integer-order global derivative, respectively. ${ᙀ}_{i}$ from i=1,2,3 are defined in Equation (2).

The piecewise derivative of a function allows us to describe the continuity of the system on each interval, eliminating any discontinuities and rendering it differentiable. Additionally, we can apply a non-singular kernel based on the Mittag–Leffler law to the second interval which generalises the derivative and acts as a global operator. This approach provides a comprehensive description of the dynamics of the system over the entire range from 0 to 1.

The objective of this model is to examine the business dynamics of a system with three agents via a piecewise integer order approach and using a global ABC operator. This enables us to understand abrupt changes and the overall density of all agents within the system. By applying this methodology, we can gain insights into the interactions of the agents and the factors that drive changes in the system. Ultimately, this analysis enables us to make more informed decisions and better manage the system holistically.

## 2. Basic Results

We discuss the fundamentals of FC in addition to the piecewise Atangana–Baleanu global operator and its antiderivative.

**Definition** **1.** *The definition of the ABC operator of the function F(t) under the condition $F\left(t\right)\in H^{1}\left(0,T\right)$ is*
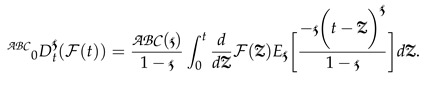
(4)*One can replace* 
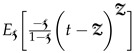
 *by* 
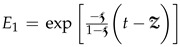
 *in (4) to obtain the Caputo–Fabrizio operator.*

**Definition** **2.** *Consider F(t)∈P[0,T], then the ABC integral is*


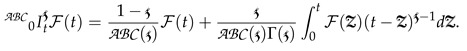

(5)

**Definition** **3** ([31])**.** *Suppose F(t) is differentiable and the increasing mapping is f(t); accordingly, the integer-order piecewise differentiation can be formulated as*

$$\begin{array}{c} {}_{0}^{PF}D_{t}F\left(t\right)=\left\{\begin{array}{cc} & F\left(t\right),\;\;0<t\leq t_{1},\\ & \frac{F^{\prime }\left(t\right)}{f^{\prime }\left(t\right)}\;\;t_{1}<t\leq t_{2}=T, \end{array}\right. \end{array}$$

*and the integration can be written as*

$$\begin{array}{c} {}_{0}^{PF}I_{t}F\left(t\right)=\left\{\begin{array}{cc} & \int_{0}^{t}F\left(\tau \right)d\tau ,\;\;0<t\leq t_{1},\\ & \int_{t_{1}}^{t}F\left(\tau \right)f^{\prime }\left(\tau \right)d\left(\tau \right)\;\;t_{1}<t\leq t_{2}, \end{array}\right. \end{array}$$

*where ${}_{0}^{PF}D_{t}F\left(t\right)$ and ${}_{0}^{PF}I_{t}F\left(t\right)$ are used for the classical derivative and integration for $0<t\leq t_{1}$, and the global derivative and integral for $t_{1}<t\leq t_{2}$, respectively.*

**Definition** **4** ([31])**.** *Let function F(t) be derivable; accordingly, the integer-order and global-order piecewise derivatives can be written as*

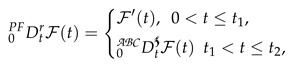

*while the integration is defined as*

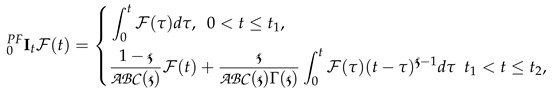

*where ${}_{0}^{PF}D_{t}^{r}F\left(t\right)$ and ${}_{0}^{PF}I_{t}F\left(t\right)$ are used for the integer-order derivative on $0<t\leq t_{1}$ and the arbitrary-order derivative and integration for $t_{1}<t\leq t_{2}$, respectively.*

## 3. Theoretical Analysis

We test whether the piecewise system solution exists, and if it does, we test whether it is unique. To this end, we express the model (2) under

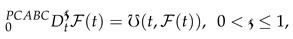

as

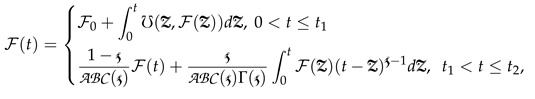
(6)
where

(7) $$F\left(t\right)=\left\{\begin{array}{cc} & Q(t)\\ & B(t)\\ & N(t) \end{array}\right.\;\;\;F_{0}=\left\{\begin{array}{cc} & Q(0)\\ & B(0)\\ & N(0) \end{array}\right.\;\;\;F_{t_{1}}=\left\{\begin{array}{cc} & Q_{t_{1}}\\ & B_{t_{1}}\\ & N_{t_{1}} \end{array}\right.\;\;\;ᙀ\left(t,F\left(t\right)\right)=\left\{\begin{array}{c} {ᙀ}_{i}=\left\{\begin{array}{cc} & \frac{d}{dt}{ᙀ}_{i}\left(Q,B,N,t\right)\\ & {}^{ABC}{ᙀ}_{i}\left(Q,B,N,t\right) \end{array}\right. \end{array}\right.,$$

where i=1,2,3.

Let $\infty >t_{2}\geq t>t_{1}>0$ with a closed norm space $E_{1}=C\left[0,T\right]$ have the norm defined as

$$∥F∥=\max_{t\in [0,T]}\left|F\left(t\right)\right|.$$

We use the following condition for the non-linear operator to achieve the results related to uniqueness and existence as

**(A1)**  ∃$L_{F}>0$; ∀$ᙀ,\;\bar{F}\in E$ we have
  $$|ᙀ\left(t,F\right)-ᙀ\left(t,\bar{F}\right)|\leq L_{ᙀ}\left|mathcalF-\bar{F}\right|,$$
**(A2)**  ∃$C_{ᙀ}>0$&$M_{ᙀ}>0,$;
  $$\left|ᙀ\left(t,F\left(t\right)\right)\right|\leq C_{ᙀ}\left|F\right|+M_{ᙀ}.$$

**Theorem** **1.** *Let G be a piecewise derivative defined on the intervals $0<t\leq t_{1}$ and $t_{1}<t\leq t_{2}$ on [0,T] that obeys (A2); accordingly, the piecewise defined derivable system (3) has a ≥1 root on the two subintervals.*

**Proof.**  By applying Schauder’s theorem of fixed point theory, we selected the close subset for both intervals of 0,T as *B* of *E* as

$$B=\{F\in E:\;∥F∥\leq R_{1,2},\;R>0\},$$

Further, we also chose a mapping T:B→B and used (6) as

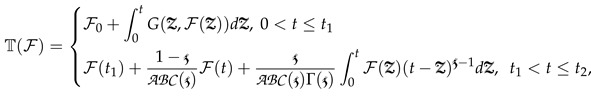
(8)
On some F∈B,

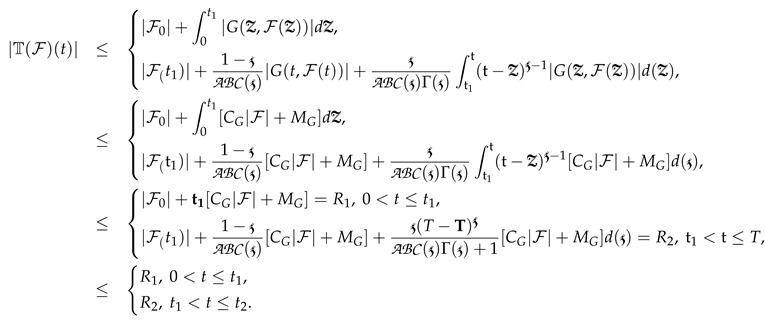

This implies that, as F∈B, T(B)⊂B. Further, this indicates that T is closed and in the given space. Moreover, for complete continuity, we process $t_{i}<t_{j}\in \left[0,t_{1}\right]$ for the 1st interval of integer derivative and assume
(9)
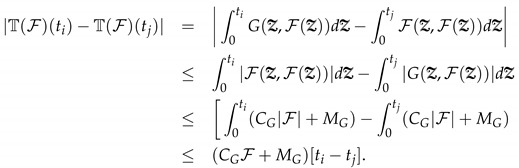

Equation (9) implies $t_{j}\to t_{i}$; accordingly,

$$|T\left(F\right)\left(t_{i}\right)-T\left(F\right)\left(t_{j}\right)|\to 0,\;\mathrm{as}\;t_{j}\to t_{i}.$$

Hence, T is equi-continuous on the $\left[0,t_{1}\right]$ set of the interval. Next, we consider the second set of interval $t_{p},t_{r}\in \left[t_{1},T\right]$ in terms of ABC as

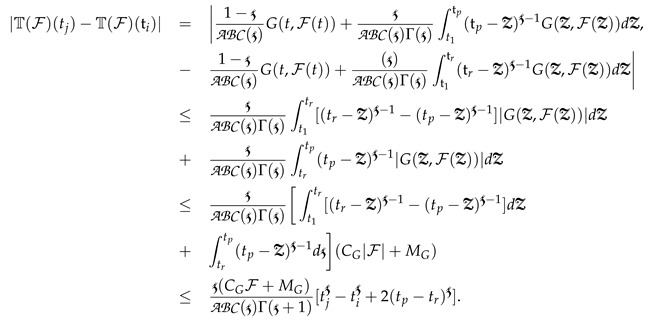
(10)
Equation (10) implies $t_{n}\to t_{m}$, and accordingly,

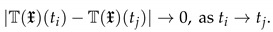

Therefore, T is equi-continuous in the $\left[t_{1},t_{2}\right]$ set of the interval, and therefore, T is the equi-continuous operator. Based on the Ascoli–Arzelá result, the operator T is completely continuous, which implies that T is continuously uniform with bounds. Finally, based on Schauder’s findings of the fixed point theory, the piecewise global derivative model (3) has at least one root on every set of partitioned intervals. □

**Theorem** **2.** *According to assumption (A1), the suggested piecewise problem has one solution if T is constructed.*

**Proof.**  Previously, we defined T:B→B to be piecewise continuous, and we considered F and $\bar{F}\in B$ on $\left[0,t_{1}\right]$ in the integer-order derivative as

(11) $$\begin{array}{ccc} ∥T\left(F\right)-T\left(\bar{F}\right)∥ & = & \max_{t\in [0,t_{1}]}|\int_{0}^{t_{1}}G\left(Z,F\left(Z\right)\right)dZ-\int_{0}^{t_{1}}G\left(Z,\bar{F}\left(Z\right)\right)dZ|\\ & \leq & t_{1}L_{G}∥F-\bar{F}∥. \end{array}$$

Equation (11) implies that

(12) $$\begin{array}{c} ∥T\left(F\right)-T\left(\bar{F}\right)∥\leq t_{1}L_{G}∥F-\bar{F}∥. \end{array}$$

In this equation, T is the contracted operator. Therefore, based on the findings of Banach’s theorem of contraction, the investigated system has one root in the set of all intervals. Furthermore, for other sets of intervals $t\in [t_{1},t_{2}]$ through the global ABC operator,

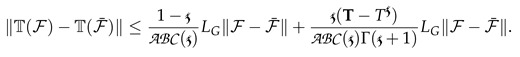
(13)
From (13), we have

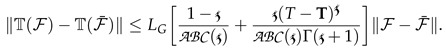
(14)
Thus, operator T is contracted. Finally, based on Banach’s contraction theorem, the suggested model has one root in the 2nd interval. Therefore, based on Equations (12) and (14), the piecewise derivative problem has one root on every set of the interval. □

## 4. Numerical Scheme

We have developed an approximate method for the piecewise global model described in Equation (3). This method involves deriving a numerical technique for each of the two sets of intervals in the context of both the integer order and ABC operator frameworks. The technique we use for the piecewise model is an integer-order technique. To apply this method to the model using Equation (3), we use a piecewise integral approach within the frameworks of the integer order and ABC operator. This approach provides a more accurate approximation of the behaviour of the system and allows us to gain valuable insights into its dynamics.

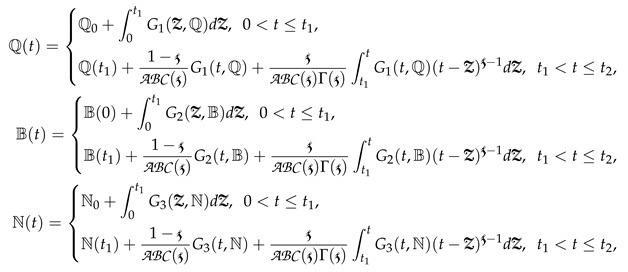
(15)
Now, we derive the numerical technique for one of the equations of problem (15), and the remaining equations are derived similarly.

At $t=t_{p+1}$,

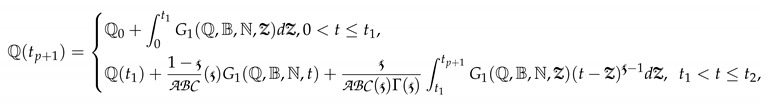
(16)


By expressing Equation (16) in the Newton scheme of interpolation from [31] as follows:

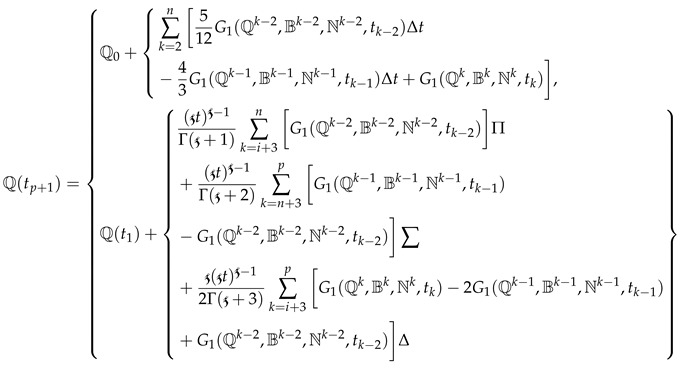
(17)


For the remaining two equations, we write the scheme as follows:

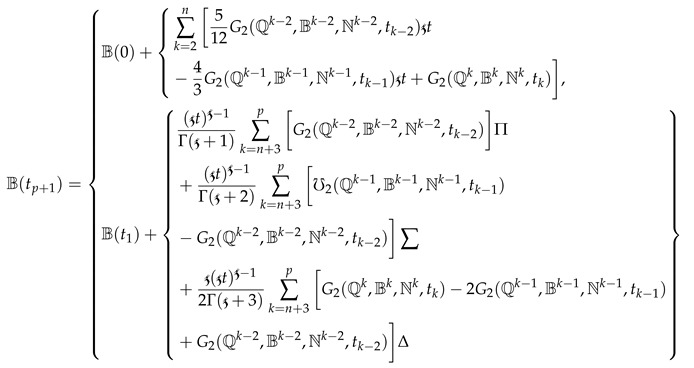
(18)

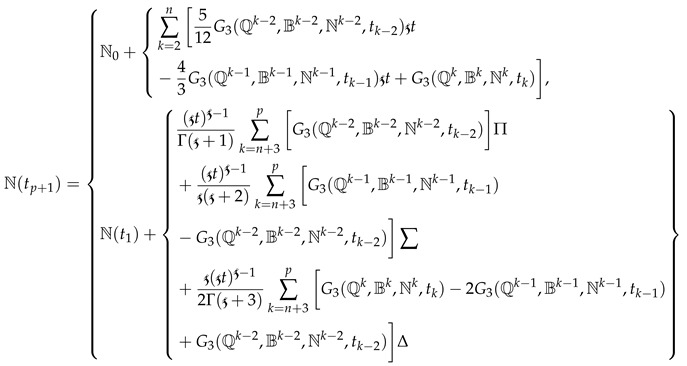
(19)
where

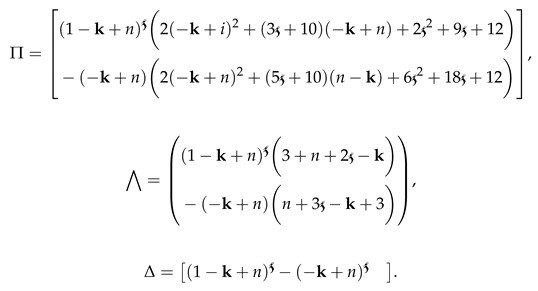



## 5. Numerical Simulation

**Figure 1:**
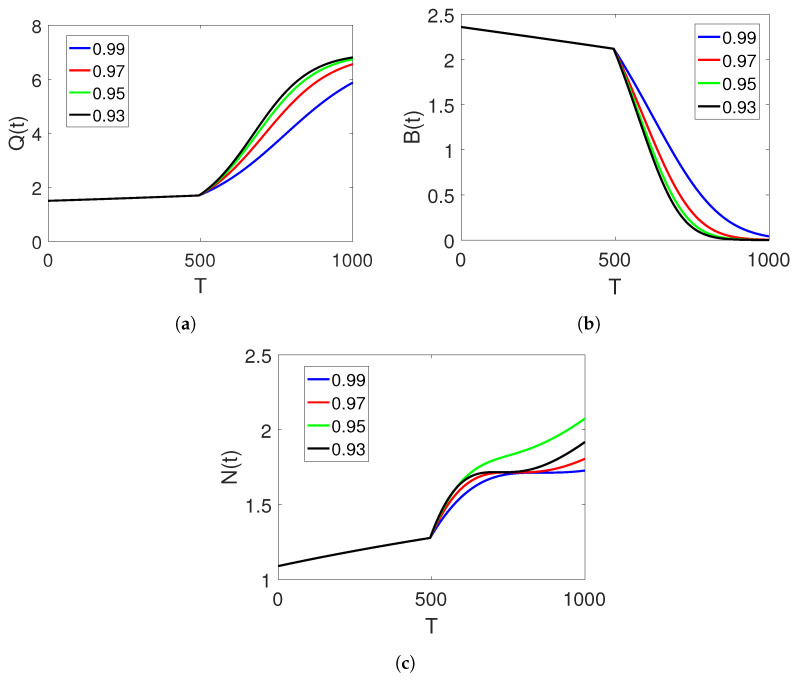
Dynamics of classes (Q(t)), (B(t)) and (N(t)) on different arbitrary orders 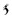 =0.99,0.97,0.95,0.93 and the time durations on the subintervals.

We have conducted various curve simulations for all three agents using the available data on both arbitrary and classical orders and time duration in days. Our simulations use the initial values of Q(0)=1.5, B(0)=2.36, and N(0)=1.09, along with the parameter values of α=0.33, δ=0.1, r=0.015, θ=0.05, and ξ=0.5556. The graphical representations are provided for the integer order and ABC operators on each interval set. Figure 1a–c depict the plots of the three classes of the proposed piecewise model for finance bubbles in the stock market on two subintervals. The first set of intervals is analysed in terms of the integer-order derivative, whereas the other interval is treated under the Atangana–Baleanu fractional operator. These show two different dynamics with bending behaviours. We observe that an increase in the shadow price of the net worth results in a decrease in the stock market bubbles and net worth quantity.

**Figure 2:**
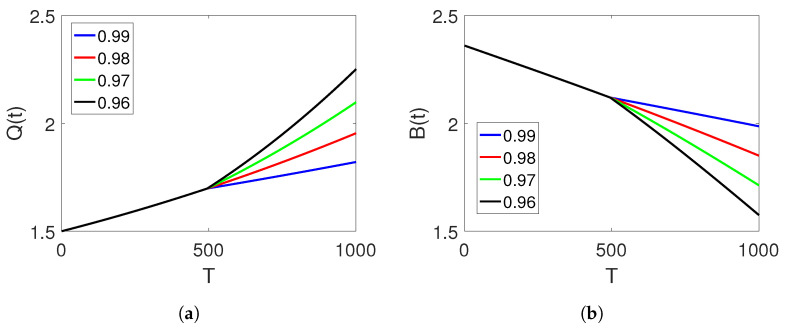

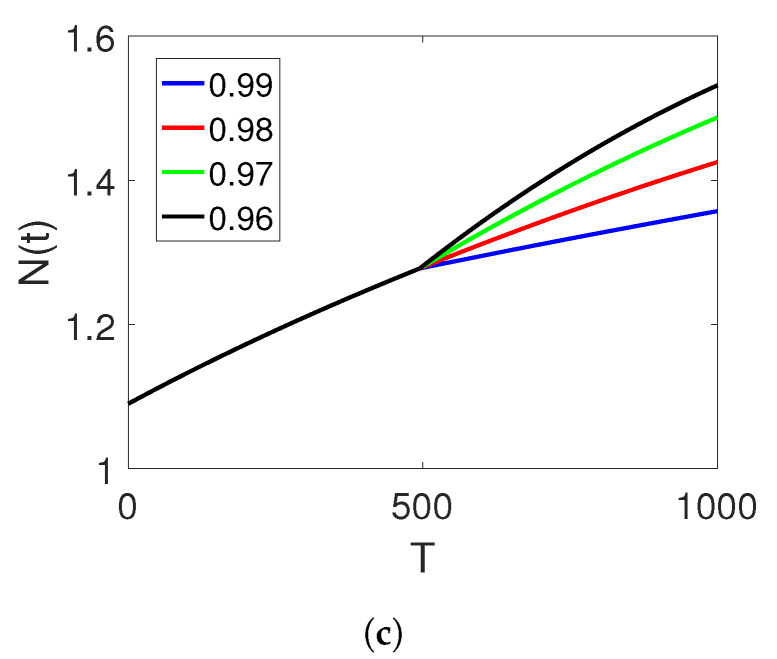
Dynamics of classes (Q(t)), (B(t)), and (N(t)) on different arbitrary fractional orders 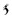 =0.99,0.98,0.97,0.96, and the time durations on two sets of intervals.

Figure 2a–c illustrate the simulation results of the three agents of the proposed piecewise model for finance bubbles in the stock market within two different subintervals. The first interval is analysed in terms of the natural-order derivative, whereas the dynamics of the other interval are considered under the Atangana–Baleanu fractional operator, displaying two different dynamics with bending behaviours. The first interval shows only natural-order dynamics on one curve, whereas the second interval shows more curve dynamics. At the beginning of the simulation, we observe a rapid increase in the shadow price of the net worth, which results in a decline in the bubbles of the stock market and the net worth quantities that tend toward zero. Here, we observe a change in the fractional orders.

**Figure 3:**
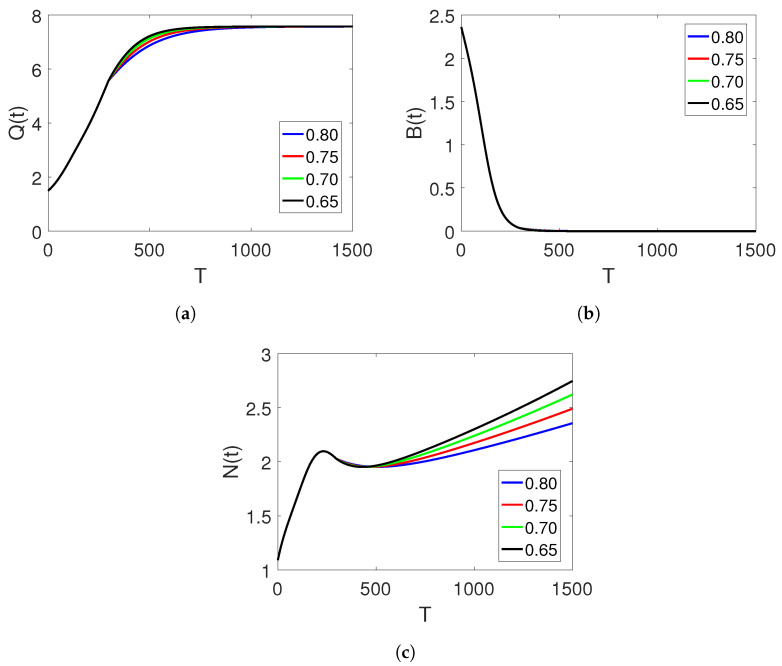
Dynamics of classes (Q(t)), (B(t)), and (N(t)) on different arbitrary fractional orders 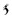 =0.80,0.75,0.70,0.65 and the time durations on the two sets of intervals.

Figure 3a–c display the dynamical behaviour of the three classes on another set of fractional orders of the proposed piecewise model for finance bubbles in the stock market on two subintervals. The first interval is analysed in terms of the natural-order derivative, whereas the dynamics of the other intervals are considered under the Atangana–Baleanu fractional operator, displaying two different dynamics with bending behaviours. In this set of data, we observe a greater increase in the shadow price of net worth, which moves towards stability. The bubbles of the stock market and the net worth quantities slightly decrease with time in this set of data and eventually become stable.

**Figure 4:**
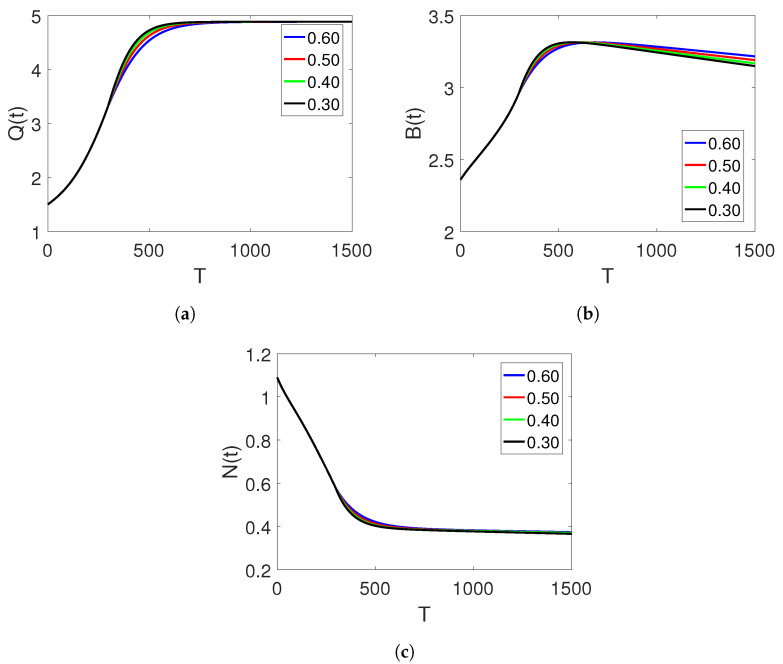
Dynamics of classes (Q(t)), (B(t)), and (N(t)) on different arbitrary fractional orders 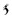 =0.60,0.55,0.50,0.45, and the time durations on two set of intervals.

Figure 4a–c illustrate the plots of the three classes for the proposed piecewise model of finance bubbles in the stock market on two subintervals of classical and fractional ABC orders while considering longer time durations and changing the order of derivatives. We observe that an increase in the shadow price of the net worth results in a decrease in the bubbles of the stock market and the net worth quantity. Stability is reached rapidly in this case.

**Figure 5:**
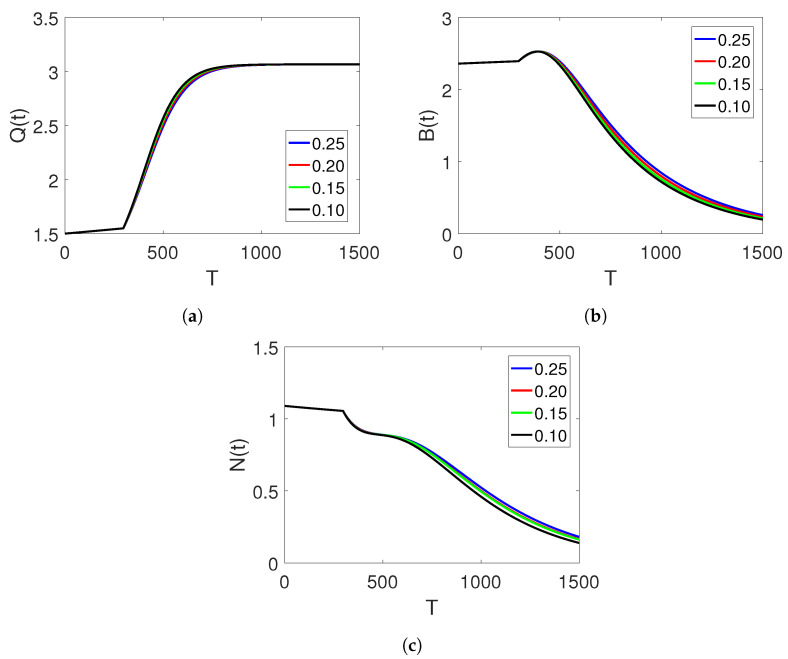
Dynamics of classes (Q(t)), (B(t)), and (N(t)) on different arbitrary fractional orders 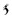 =0.25,0.20,0.15,0.10, and the time durations on two sets of intervals.

Figure 5a–c show the plots of the three classes based on a change in the value of fractional orders to small fractional orders of the considered piecewise model for the finance bubbles of the stock market on two subintervals showing the sensitivity of the fractional order parameter 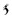.

**Figure 6:**
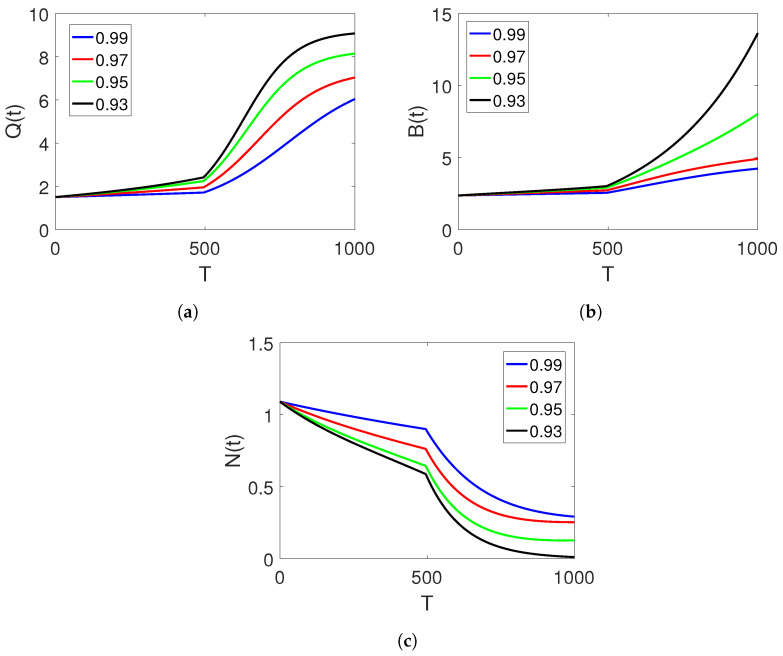
Dynamics of classes (Q(t)), (B(t)), and (N(t)) on different arbitrary fractional orders 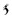 and the time durations on the two set of intervals.

Figure 6a–c show the plots of the three classes by changing the value of δ=0.01 for the proposed piecewise model for the finance bubbles of the stock market on two subintervals, showing the sensitivity of the said parameter on the same fractional orders as before. Here, we consider both intervals as fractional orders.

**Figure 7:**
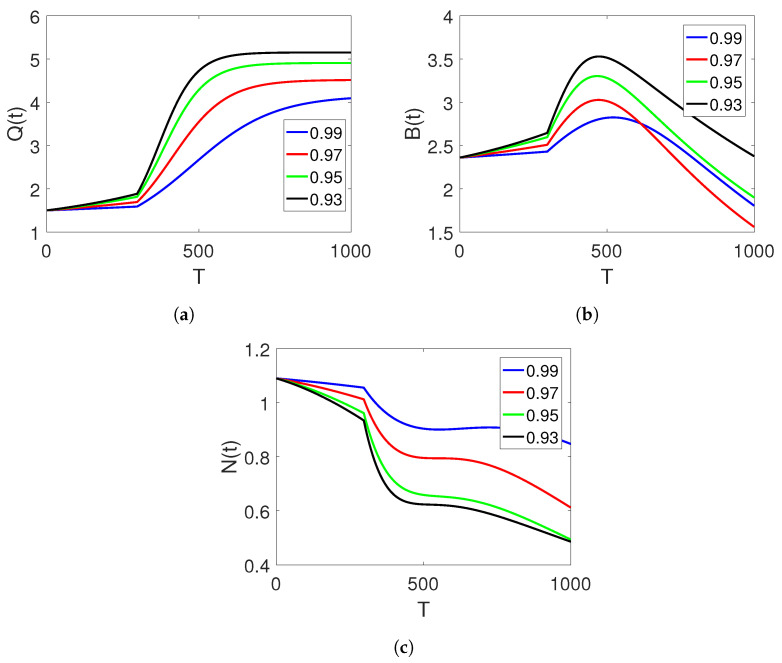
Dynamics of classes (Q(t)), (B(t)), and (N(t)) on different arbitrary fractional orders 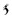, and the time durations on the two set of intervals.

Figure 7a–c show the three classes of the proposed piecewise model for the finance bubbles of the stock market on two subintervals. The first interval is treated under the Caputo fractional derivative, and the other interval is treated under the ABC derivative. For the sensitivity of the parameter *r*, we change the value of *r*.

**Figure 8:**
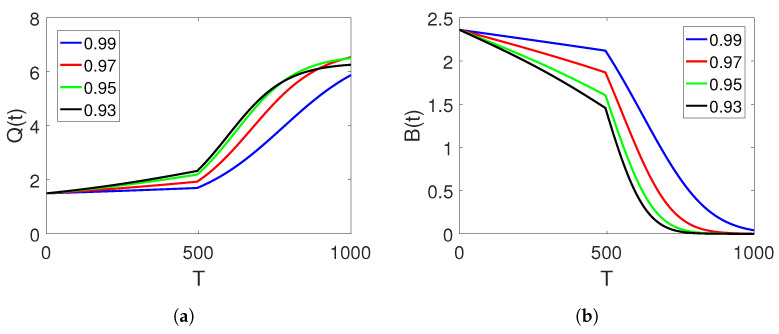

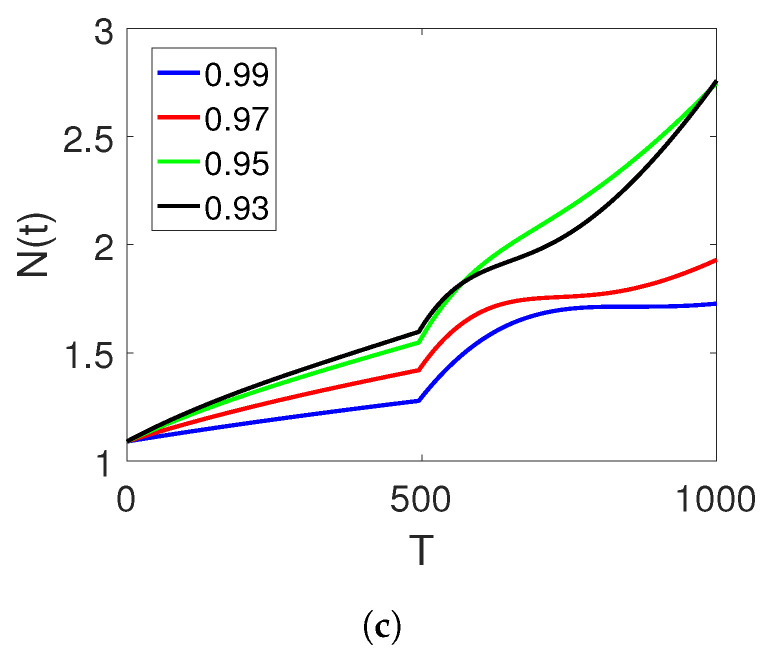
Dynamics of classes (Q(t)), (B(t)), and (N(t)) on different arbitrary fractional orders 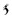 and the time durations on the two set of intervals.

Figure 8a–c show the results of the three classes of the proposed piecewise model for the finance bubbles of the stock market on the two subintervals. Here, the first interval is tested in the Caputo fractional derivative, and the other interval is used for the ABC derivative. To address the sensitivity of parameter ζ, we change the value of ζ.

## 6. Conclusions

This paper presents a novel framework of piecewise global derivatives, which has been successfully applied to the analysis of financial bubbles in the stock market. The problem is formulated using the integer order and Atangana–Baleanu derivative on two sets of partitioned intervals, resulting in piecewise dynamics. Through a fixed point theory analysis, the existence and uniqueness of the considered problem are established for each interval. A numerical solution method is developed based on the interpolation of Newton’s polynomial in an integer order derivative for each sub-interval and the Atangana–Baleanu operator for global orders. The fractional order derivative parameter in the numerical scheme provides a continuous and density spectrum of all financial bubble agents. The numerical simulation of the total compartments provides six data points, and the piecewise influences on $t_{1}$ are analysed. The first interval is simulated in the integer order, whereas the second interval is tested at various fractional orders. All curves converge to the integer order as the derivative order increases. This type of development can be applied to various global phenomena where sudden variations occur in the dynamics of different densities, and the instability can be stabilised by dividing the time interval into two or more intervals. The study shows how shadow prices can be controlled by including various financial bubbles in the stock market to affect the net worth, demonstrating the potential applications of the framework. The control strategy is implemented more effectively on smaller fractional orders. Therefore, through this analysis, we can manage the variability in shadow prices using piecewise analysis. This is because non-linear and piecewise derivative systems can account for most of the irregular activities and dynamics.

## Data Availability

Not applicable.
